# An efficient protoplast-based genome editing protocol for *Vitis* species

**DOI:** 10.1093/hr/uhad266

**Published:** 2023-12-13

**Authors:** David M Tricoli, Juan M Debernardi

**Affiliations:** Plant Transformation Facility, University of California, Davis, CA 95616, USA; Plant Transformation Facility, University of California, Davis, CA 95616, USA

## Abstract

CRISPR-Cas technologies allow for precise modifications in plant genomes and promise to revolutionize agriculture. These technologies depend on the delivery of editing components into plant cells and the regeneration of fully edited plants. In vegetatively propagated plants, such as grape, protoplast culture provides one of the best avenues for producing non-chimeric and transgene-free genome-edited plants. However, poor regeneration of plants from protoplasts has hindered their implementation for genome editing. Here, we report an efficient protocol for regenerating plants from protoplasts from multiple grape varieties. By encapsulating the protoplasts in calcium alginate beads and co-culturing them with feeder cultures, the protoplasts divide to form callus colonies that regenerate into embryos and ultimately plants. This protocol worked successfully in wine and table grape (*Vitis vinifera*) varieties, as well as grape rootstocks and the grapevine wild relative *Vitis arizonica*. Moreover, by transfecting protoplasts with CRISPR-plasmid or ribonucleoprotein (RNP) complexes, we regenerated albino plants with edits in *VvPHYTOENE DESATURASE* gene in three varieties and in *V. arizonica*. The results reveal the potential of this platform to facilitate genome editing in *Vitis* species.

## Key message

An integrated cell biology platform allows for the development of embryogenic callus, cell suspension, protoplast culture, transfection and regeneration of genome-edited *Vitis* plants.

## Introduction

Plant transformation and genome editing are excellent tools for crop improvement and basic research. However, in many crops, the potential of transgenesis and genome editing is limited by the low transformation ability of most cultivars and the intensive labor and space required to supply adequate tissue for transformation [1]. In grapevines, protocols have been developed for *Agrobacterium tumefaciens*-mediated transformation of table grapes [2], rootstock cultivars [3], and wine grapes [4]; however, transformation efficiencies remain low, is highly genotype dependent, and the time required to generate transgenic plants is lengthy.

**Figure 1:**
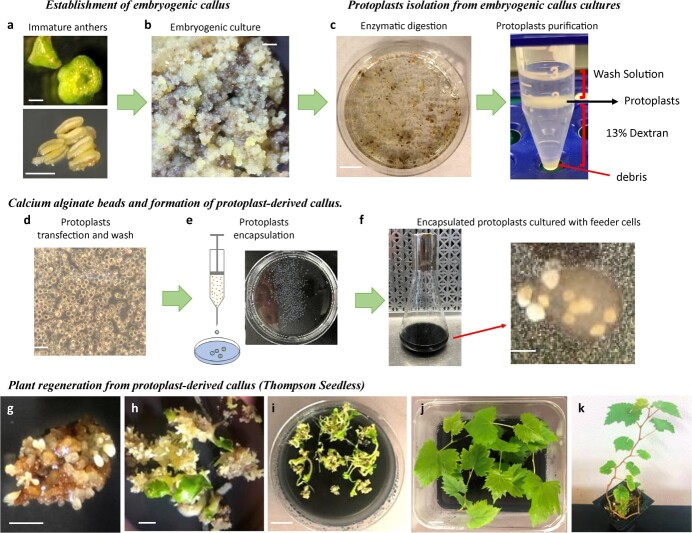
An integrated protocol for embryogenic callus induction, protoplasts isolation and culture, and plant regeneration in *Vitis vinifera* varieties. **a.** Establishment of embryogenic calli from immature anthers. Immature flowers from table grape Thompson Seedless harvested in early to mid-April in northern California, surface-sterilized and cut to release immature anthers. Scale = 1 mm. **b.** Immature anthers cultured in embryogenic induction media for 12–16 weeks differentiate into embryogenic calli. Scale = 1 mm. **c.** Embryogenic callus samples treated with enzymatic solution in petri dishes to digest the cell wall and release protoplasts. The protoplasts are them purified using a dextran gradient. Scale = 1 cm. **d.** Isolated Thompson Seedless protoplasts. At this stage, protoplasts can be transfected with plasmid DNA or RNP complexes. Scale = 100 μm. **e.** Encapsulation of Thompson Seedless protoplasts in alginate beads. Using a sterile syringe, the protoplast/alginate solution is expelled dropwise into a petri dish containing an osmotically adjusted 50 mM CaCl_2_ solution. **f.** Encapsulated protoplasts are cultured in liquid media in the presence of feeder cells. After 42–56 days in liquid media, encapsulated protoplasts form isolated small callus colonies. Scale = 1 mm. **g–k.** Plant regeneration from protoplast-derived callus colonies. Alginate beads containing Thompson Seedless callus colonies transferred to germination media (**g**), where calli differentiate into embryos that germinate out of the beads (**h**), scales = 1 mm. Each bead generates multiple embryos that continue to develop into fully formed seedlings (**i**), scale = 1 cm. Individual seedlings are transferred to larger containers (**j**, scale = 1 cm) where they continue development and are later transferred to soil (**k**).

The use of genome editing technologies to improve vegetatively propagated crops, like grapevines, has other challenges. In seed-propagated crops, edited genes can be segregated out of the transgenic population by breeding to focus only on the desired gene edits [5]. The segregation step also helps to fix CRISPR-induced mutations in the following generations, as the first generation T0 plants are frequently biallelic, heterozygous, or chimeras with multiple mutations. However, for clonally propagated grapevines (and many other perennials), it is not possible to use breeding to eliminate CRISPR-Cas9 sequences, fix mutations, and maintain the fidelity of clonal germplasms. A limited number of grapevine clones have been used for many decades to produce high-quality wine. These clones are maintained by vegetative propagation to preserve the intrinsic quality of these materials. Therefore, the implementation of genome editing technology to introduce new traits into existing *Vitis vinifera* varieties without altering their essential characters and identity is crucial.

Compared to *Agrobacterium*-mediated transformation of embryogenic callus, protoplast culture provides a viable avenue for producing non-chimeric and transgene-free genome edited plants. Cas9-gRNA ribonucleoprotein (RNP) complexes have been introduced into plant protoplasts using particle bombardment, polyethylene glycol (PEG)-mediated transfection, lipofection, or electroporation to edit the genome without integration of exogenous CRISPR-Cas9 DNA [6]. Protoplasts reform cell walls within 48–72 hours and the edited cells can be stimulated to form callus colonies. Although protoplast-mediated genome editing is a very promising technology for grapevines, routine regeneration of whole plants from transfected protoplasts for a wide range of grape genotypes has not previously been achieved in grape [7–12]. Xu et al., 2007 demonstrated successful isolation of protoplasts from grapes; however, the ability of protoplasts to divide and produce callus was very low, with <5% of the isolated protoplasts forming calli. More recently, two works have reported successful regeneration of plants from transfected protoplasts [13, 14], yet when those protocols were tested in a few table grape varieties, the number of regenerated plants was still low [13, 14], and may require further optimization for other élite cultivars, including wine varieties, as indicated by the authors [14].

Here we report an efficient protocol for isolating protoplasts from embryogenic callus cultures and inducing them to regenerate into plants. The whole protocol is fast, requiring 6 months, works in multiple grape varieties, and we could efficiently utilize it with RNP genome editing technology to create genome-edited plants from table and wine grape varieties and a wild relative.

## Results

Over the last 10 years, we have developed extensive cell biology capabilities in grape, which include the establishment of embryogenic calli from anthers and regeneration of whole plants from somatic embryos (Fig. 1). We have utilized these methodologies to develop a protocol that allows for the successful isolation, purification, and culture of protoplasts from embryogenic cultures generated from the table grape variety, Thompson Seedless (Fig. 1a-c). In this protocol, isolated protoplasts are encapsulated in calcium alginate beads [15] and co-cultured with cell suspension feeder cultures generated from the grape rootstock cultivar 1103P (*Vitis berlandieri × Vitis rupestris;* Federico Paulsen, 1896), which have lost their ability to regenerate into embryos or plants (Fig. 1d–f). We found that the co-cultivation step with an active growing cell suspension culture is critical to induce protoplast division and calli formation, as protoplasts failed to divide and form callus colonies in the absence of feeder suspensions (Table S1).

**Figure 2:**
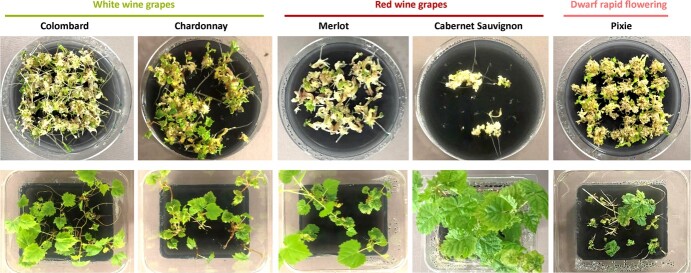
Plant regeneration from protoplast-derived calli from multiple wine grape varieties. Top: germinating embryos and developing seedlings from encapsulated protoplasts in germination media from white and red wine varieties. Bottom: fully developed plants in larger containers before being acclimated in soil.

In the presence of feeder cultures, encapsulated protoplasts began dividing at ~7–14 days. By day 21 postencapsulating, we could observe small callus colonies, which continued to develop within the calcium alginate beads and often grew large enough that we could see them rupturing through the surface of the beads (42–56 days postencapsulating) (Fig. 1f). A subset of calcium alginate beads was then transferred onto agar-solidified medium (germination media), where embryogenic callus colonies began producing somatic embryos. After ~80 days, we observed embryos germinating out of the beads, which were able to develop into whole seedlings (Fig. 1g–i). Individual seedlings were then transferred to larger containers and continued to grow into fully developed plants, which were later moved to soil (Fig. 1j–k). Using this protocol, we were able to regenerate a large number of plants, in the order of hundreds. Starting with 0.5–1 g of embryogenic calli, we isolated 5–25 million protoplasts and encapsulated 1 million in 400 alginate beads that on average contained 2500 protoplasts. After co-cultivation with feeder cells, we observed that each alginate bead contained ~150 calli colonies, indicating that ~7% of encapsulated protoplasts were able to generate calli colonies (Table S1 andS2). Note that in these experiments, only a subset of alginate beads (5%–10%) was transferred to germination media, as we normally plated 25 beads per plate. Once on germination media, callus colonies differentiate into embryos that germinate, with individual beads producing 0–10 seedlings. We found it difficult to estimate the percentage of calli colonies fully regenerating into plants, as we have observed that germinating embryos could produce secondary embryos that also regenerate into plants. Instead, we focused on the number of independent alginate beads generating seedlings, which was close to 100% for Thompson Seedless (Table S3) and considered plants coming from the same beads as potential clones. We tracked the bead from which each plant originated. Therefore, although it is possible that plants coming from the same bead might be clones, plants harvested from different beads or different plates must be independent events. The regenerated plants showed normal morphology and were indistinguishable from clonally propagated plants (Fig. 1k). The whole protocol, from protoplast isolation to recovery of plants, takes ~6 months.

Plant regeneration can be highly genotype dependent, which is a critical barrier in several crops and often restricts the application of genome editing technology to only a few cultivars. Therefore, we next tested whether the protocol we developed in Thompson Seedless was applicable to a wider range of grape varieties (Fig. 2). We generated embryogenic calli from anthers of wine varieties Cabernet Sauvignon, Chardonnay, Colombard, and Merlot. We also used a rapid-flowering dwarf grape variety, named Pixie, which was derived from the champagne red wine variety Pinot Meunier [16]. In addition, we tested rootstocks 101–14 (*Vitis riparia* × *V. rupestris* hybrid; Millardet and de Grasset, 1882) and UC-GRN1 (*V. rupestris* × *Muscadinia rotundifolia* hybrid [17]), and the wild relative *V. arizonica*, which has been used to breed grape cultivars resistant to Pierce’s Disease [18].

Once embryogenic calli were established for all cultivars, we tested protoplast isolation, encapsulation, and plant regeneration. Although we observed differences in protoplast yields between genotypes, all of them could form callus colonies upon encapsulation. Importantly, after transferring the beads to germination plates, we observed that all varieties were able to regenerate into seedlings and fully developed plants (Fig. 2, Fig. S1). We did notice differences in the number of beads producing embryos that germinated and developed into full plants between varieties, with Colombard being as efficient as Thompson Seedless; Pixie, Chardonnay, Merlot, GRN1, and *V. arizonica* showing intermediate efficiency; and Cabernet Sauvignon and the rootstocks 101–14 being less efficient (Fig. 2, Fig. S1, Table S3).

Once we had established a protoplast-based regeneration protocol that yielded a large number of plants, we tested to see if we could use it to generate edited plants by transfecting plasmid DNA encoding a CRISPR-Cas9 editing machinery. In one experiment, we transfected Thompson Seedless protoplasts with a vector having two gRNAs targeting sequences in exons 5 and 7 of the *VvPHYTOENE DESATURASE* (*VvPDS*, *GSVIVT01016650001*) gene (Fig. S2a, b), which when inactivated generate albino plants [11, 19]. Protoplasts were encapsulated after transfection, grown in a medium containing grape feeder suspension, and developed callus colonies within the alginate beads. We performed two transfections, and from each of them we plated 25 alginate beads in germination plates. We visually screened the regenerated seedlings from independent beads and identified three independent albino seedlings (Fig. S2c). After DNA isolation and amplicon sequencing, we confirmed that these three lines contained editing events in the targeted regions in both chromosomes that likely disrupt *VvPDS* gene function (Fig. S2b).

**Figure 3:**
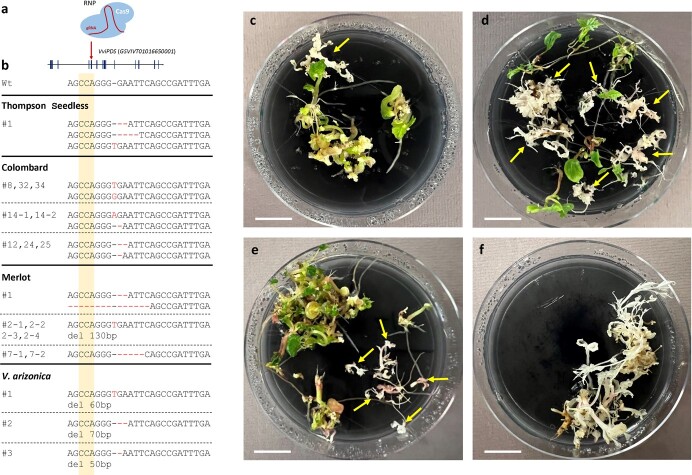
Regeneration of *PDS*-edited grape plants from RNP-transfected protoplasts. **a.** Transfection of protoplasts with Cas9-gRNA RNP complexes targeting a sequence in the fifth exon of grape *PDS* (*GSVIVT01011710001*) gene. **b.** Sequences of the targeted site in the fifth exon of *PDS* in wild-type (Wt) and albino plants from Thompson Seedless, Colombard, and Merlot varieties and *V. arizonica*. The PAM sequence is indicated in yellow. Insertions are indicated in red letters, while deletions as red dashes. Large deletions are indicated as del 50 bp, del 60 bp, del 70, and del 130 bp. Sequences were obtained by amplicon sequencing. We tracked each shoot to the beads it was originated with a number #. Colombard lines #14–1 and #14–2 have the same genotype and originated from bead #14. Similarly, Merlot lines #2–1,2–2, 2–3, and 2–4, which originated from bead #2. These events are likely clones. **c-f.** Plates showing regenerated seedlings from RNP-transfected protoplasts from different grape varieties. *PDS* knockout seedlings showing albino phenotype are indicated with yellow arrows. **c**, Thompson Seedless; **d**, Colombard; **e**, Merlot; **f**, *V. arizonica*. Scale = 1 cm.

In another experiment, we targeted the DELLA domain of the *VvGAI1* gene (*GSVIVT01011710001*). An amino acid change in this domain was reported to generate a gibberellin (GA)-insensitive allele that result in dwarf grape plants [20]. We designed gRNAs targeting six different regions along the sequence coding for the DELLA domain and cloned them in three vectors in different combinations (vector #1 contained gRNAs 1,3 and 5; vector #2 contained gRNAs 2, 4 and 6; and vector #3 gRNA 1 and 6; Fig. S2d). The three vectors were cotransfected into Thompson Seedless protoplasts. After plant regeneration, we isolated genomic DNA from root tissue from 90 randomly selected seedlings. Analysis of the sequencing results revealed editing events in five regenerated plants (Fig. S2e). Four of those lines (#21133–2-3, #21133–5-1, −5, and − 6) showed a severely dwarfed phenotype *in vitro* (Fig. S2f). Line #21133–2-3 was heterozygous harboring a mutant allele with an extra T (Allele-T) in the region targeted by gRNA 6, while lines #21133–5-1, −5, and − 6 contained large deletions between gRNAs 1 and 6 that completely removed the sequence encoding the DELLA domain (Allele-1 (−76 bp), −2 (−78 bp), −3 (−77 bp)). The deletion of 78 bp in Allele-2 in plants #21133–5-1, −5, and − 6 maintains the reading frame but eliminates 26 amino acids corresponding to the DELLA domain. That mutation would result in a GA-insensitive protein that could be associated with the severe dwarfed phenotype observed in these plants.

The fifth plant with edits was regenerated from experiment 21 119 (T0#21119–1-5). This plant was heterozygous, harboring two different mutant alleles. Allele-4 contains multiple mutations distributed in the targeted regions of all six gRNAs, while the other allele (Allele-5) contains a large deletion (−77 bp) between gRNAs 1 and 6. Both mutations result in frameshifts that generate premature stop codons. Contrary to the other four dwarf lines, this line had a normal phenotype *in vitro*, and we were able to generate multiple clonal plants from this line, which were acclimated in soil in a greenhouse (Fig. S2g).

As DNA-free editing is the goal for clonally propagated crops, we tested whether we could recover edited plants after transfecting protoplasts with Cas9-gRNA RNP complexes. We transfected protoplasts from Thompson Seedless, Colombard, and Merlot varieties, and the wild relative *V. arizonica* with RNP complexes consisting of Cas9 protein and a gRNA targeting the exon 5 of *VvPDS* gene (Fig. 3a, b). For Thompson Seedless, we plated 25 alginate beads on germination medium, and all of them produced shoots, of which one showed albino phenotype (Fig. 3c). For Colombard, we performed two transfections. For replicate 1, we plated 75 beads on germination medium, and 73 produced seedlings, from which we identified 45 plants with albino phenotype (Fig. 3d). For replicate 2, we plated 25 beads on germination medium, all of which produced seedlings, from which we recovered 10 albino plants. For Merlot, we plated 25 beads, and eight beads germinated seedlings, from which we recovered 11 albino plants (Fig. 3e). Finally, for *V. arizonica,* from 75 beads plated onto germination medium, 56 produced seedlings, from which we identified five albino plants (Fig. 3f). We then isolated genomic DNA from a subset of albino plants (one plant from Thompson Seedless, 23 from Colombard, eight from Merlot, and five *V. arizonica*) and genotyped them by amplicon sequencing. The results revealed several different edits in the *VvPDS* gene in the albino plants for the four varieties (Fig. 3b, Fig. S3), which included insertions of 1 nt or deletions of 1–130 nts. Importantly, except for the albino Thompson Seedless plant that contains three alleles, all the other genotyped plants were biallelic or homozygous, indicating that the regenerated edited plants were not chimeric. It should be noted that, as we only sequenced albino shoots, heterozygous editing events containing an edited copy and a wild-type copy of *VvPDS* were not identified. From the 23 Colombard albino plants, we could identify 14 different genotypes, while the eight Merlot plants could be grouped into three different genotypes and the five *V. arizonica* into four genotypes. The lines with the same genotype could be independent events producing the same genotype or could be clones derived from a single editing event, like Colombard lines #14–1 and #14–2, which germinated from the same bead.

## Discussion

Our results show that the ability to regenerate plants from protoplasts of multiple grape genotypes offers an avenue to deploy genome editing techniques to grapevine using plasmid DNA as well as RNP-based genome editing approaches. Using RNP complexes eliminates any possibility of integration of foreign DNA into the plant genome, thereby allowing the generation of non-transgenic genome-edited plants. We envision that this platform could also be combined with newer and more precise genome editing approaches [21], whose efficiency is lower than regular CRISPR-Cas9 knockout and require screening a large number of events.

Transfection and editing of protoplasts using RNPs, including grape protoplasts, has been reported in the literature [6, 13, 14]. Nevertheless, regeneration of whole plants from protoplasts has been a major roadblock to implementing genome editing technology in grapes and remains so for many other vegetatively propagated plants. The protocol described here allows for the successful isolation of protoplasts from embryogenic cultures, the formation of protoplast-derived callus, and whole-plant regeneration in multiple germplasms. The regenerated plants from different varieties showed normal morphology and were indistinguishable from other *in vitro*-propagated plants. In this protocol, embryogenic calli were generated using immature anthers, so there is a possibility that plants regenerated could be haploid. However, we observed that in our system, callus develops from the anther filaments, which is a somatic tissue, and we carefully selected anthers in a premeiotic stage, which would minimize the chances of getting haploid plants. This is supported by the high frequency of biallelic-edited plants recovered. Still, we cannot rule the possibility of somaclonal variations generated during protoplast culture. Indeed, plants regenerated from protoplasts have been found to have chromosomal instability even though the plants appeared phenotypically normal [22]. We think that our protocol, which allows for the production of a large number of plants significantly increases the probability of identifying edited plants with true-to-type phenotypes.

Compared with other protocols [8, 13, 14], this regeneration protocol results in hundreds of regenerated plants from single experiments. We found that in our hands, co-cultivation with feeder suspension cells was critical to stimulate the isolated protoplasts to divide efficiently and form callus colonies that eventually regenerate into embryos and ultimately whole plants. In recently published protocols, other groups have been able to regenerate table grape-variety plants without feeder suspensions, indicating that modifications in media composition or incubation conditions can also induce protoplast division. However, our results indicate that the presence of feeder significantly improves the process, which is critical when working with more difficult varieties. We also think that a better understanding of the regeneration mechanism promoted by the feeder cultures would allow the design of simpler or even more efficient platforms in the future.

Encapsulating protoplasts in alginate beads is critical, as the beads provide a barrier to separate protoplasts from feeder cells, which helps avoid contamination with feeder cells during the regeneration step. In addition, the bead matrix prevents the protoplasts from aggregating, which allows recovery of plants from single-cell descent, minimizing the chances of regenerating chimeric plants. In agreement with this, we observed that most genotyped edited plants were biallelic or homozygous.

Importantly, the protocol showed limited genotype dependency and worked efficiently in multiple grapevine varieties, including wine (Chardonnay, Colombard, Merlot, Cabernet Sauvignon), table (Thompson Seedless), and rootstocks (101–14, GRN1) grapes as well as the rapid-flowering dwarf grape genotype, Pixie. Successful editing of Pixie grape will allow for rapid evaluation of fruit trait-associated genes since this genotype produces fruit within 12 months [16, 20]. Moreover, we have shown successful regeneration of edited plants from protoplast of *V. arizonica,* which provides an opportunity to perform genome editing in wild relatives of grapevine that has the potential to contribute to crop improvement [23].

Although specific tissue culture methodologies are normally not directly translatable between species, the use of feeder cultures to stimulate protoplast division could be beneficial in protoplast culture of other crops. Indeed, we first observed the ability of feeder suspensions to enhance protoplast division in *Glycine max* [15], and this work was the rationale for testing feeder suspensions in grape.

Finally, we believe that the combination of this protoplast-based genome editing platform for multiple grape varieties with the existing genomic resources generated for multiple *Vitis* species [24, 25] will allow a faster characterization of agriculturally relevant traits and to implement genome editing strategies to modulate or rewire existing genetic networks to accelerate grapevine breeding in the face of climate change.

## Materials and methods

### Establishment of grape embryogenic callus

We collected immature flowers in the spring (early to mid-April in northern California) and surface-sterilized them by submersing in 1.2% sodium hypochlorite plus 5 μl tween 20 for 20 minutes. We cut the surface-sterilized flowers at the junction between the immature petals and the pedicel using a scalpel and gently squeezed the petals to release the anthers (Fig. 1a). We plated the isolated anthers on embryogenic callus inducing medium (EIM, see below) and incubated in the dark at 26°C without subculturing until calli form. Embryogenic callus developed from the anther filaments in 12–16 weeks (Fig. 1b).

Different varieties require slightly different EIM media composition to induce callus formation.

EIM media for 101–14, Cabernet Sauvignon, Chardonnay, Merlot, and Thompson Seedless (PIV [26]): agar-solidified Nitsch and Nitsch minimal organics medium [27] supplemented with 60 g/l sucrose, 4.5 μM 2, 4-dichlorophenoxyacetic acid (2,4-D), and 9 μM benzylaminopurine (BAP).

EIM media for Colombard, GRN1, and *V. arizonica* [28]: agar-solidified Murashige and Skoog minimal organics medium (MS, [29]) supplemented with 20 g/l sucrose, 4.5 μM 2,4-D, and 4.5 μM BAP.

EIM media for Pixie (PM [30]): agar-solidified major elements [27], minor elements, Fe-EDTA [29], vitamins [31], 60 g/l sucrose, 2.5 μM 2,4-D, 5 μM N-(2-chloro-4- pyridyl)-n´-phenylurea (4CPPU), 2.5 μM 2-napthoxyacetic acid (NOA), and 8 g/l phytoagar, pH 5.7.

Once calli formed, we transferred the embryogenic callus along with developing somatic embryos to Pic/TDZ medium [Lloyd and McCown Woody Plant Medium (WPM) [32] supplemented with 20 g/l sucrose, 1 g/l casein, 1 mM 2-(N-morpholino) ethanesulfonic acid (MES), 500 mg/l activated charcoal, 42 μM picloram, 9 μM thidiazuron (TDZ) solidified with 8 g/l phytoagar] and incubated at 26°C in the dark. We subcultured calli to fresh medium of the same formulation every 4 weeks.

### Protoplast isolation from embryogenic callus cultures

For protoplast isolation, we harvested ~500–1000 mg of embryogenic callus maintained on agar-solidified plates containing Pic/TDZ medium and resuspended it in 10 ml of enzyme solution [ES: Filter-sterilized 1.0% Onozuka Cellulase RS, 1.0% Onozuka Cellulase R10, 0.25% pectinase, 0.25% macerozyme R10, 0.6 M mannitol, 5 mM CaCl_2_, 10 g/l bovine serum albumin (BSA), 5 mM MES, and 3 g/l glycine, pH 6.0] in a 100 × 25-mm petri dish. We incubated the solution in the dark at 25°C on a platform shaker at 40 rpm. After ~16–24 hours incubation, we filtered the protoplast solution through a 40-μm screen and collected the protoplasts by pelleting via centrifugation at 500 g for 8 minutes. We washed the protoplasts twice in 4 ml of osmotically adjusted wash solution [FW: 0.6 M mannitol, 2 mM CaCl_2_, 1 g/l BSA, 1191 mg/l 4-(2-hydroxyethyl)-1-piperazineethanesulfonic acid (HEPES), and 40 mM glycine]. We purified protoplasts using a dextran gradient consisting of 1.5 ml of a 13% dextran solution, overlaid with 1 ml of FW solution. We could readily harvest the protoplast band from the interface between the dextran and FW solution, and we transferred them to a 60 × 15-mm petri dish using a Pasteur pipette. Yields of protoplasts from 500 to 1000 mg fresh weight of embryogenic callus ranges from 0.5 to 2.5 × 10^7^ cells per ml depending on the quality of the embryogenic callus and the genotype.

### Establishment of conditioned feeder suspensions of the grape rootstock genotype 1103P

We harvested ~1000 mg of embryogenic calli from grape rootstock genotype 1103P and transferred to 20 ml of liquid Pic/TDZ [WPM supplemented with 20 g/l sucrose, 1 g/l casein, 1 mM MES, 2 g/l activated charcoal, 0.6 mM ascorbic acid, 0.7 mM citric acid, 0.4 mM reduced glutathione, 42 μM picloram, and 9 μM TDZ, pH 5.7] in 125-ml shake flasks on a gyratory shaker at 90 rpm in the dark at 26°C. Osmotically conditioned feeder suspensions were generated by gradually increasing the osmotic potential of the suspension medium over time. During the biweekly subcultures, we removed 10 ml of the suspension and replaced it with an equal volume of liquid 0.4 M Pic/TDZ medium [WPM supplemented with 20 g/l sucrose, 42 μM picloram, 9 μM TDZ, 0.4 M mannitol, 222 mg/l CaCl_2_, 1 g/l casein, 5 mM HEPES, 0.6 mM ascorbic acid, 0.7 mM citric acid, 0.1 mM reduced glutathione, and 2 g/l activated charcoal, pH 5.7]. We repeated this process (6 times) so the cells gradually acclimated to the high-osmotic medium over time. Once acclimated to growing on high-osmotic medium, we maintained the suspension cultures by transferring 4–5 ml of a 7-day-old suspension to an empty 125-ml shake flask and added 20 ml of 0.4 M Pic TDZ medium.

### Culture grape protoplasts in calcium alginate beads stimulate the formation of protoplast-derived callus

To encapsulate protoplasts in alginate beads, we first adjusted the protoplast density to 1 × 10^6^ protoplasts in 1 ml of FW solution. Then, the protoplast solution was mixed with an equal volume of 3.2% sodium alginate solution [SAS: 72.87 g/L mannitol, 2 mM CaCl_2_, 5 mM HEPES, and 3.2 g/l sodium alginate, pH 5.7]. We drew the protoplast/alginate solution into a 12-ml sterile syringe and expelled it dropwise through a 30-gauge needle into 20 ml of an osmotically adjusted 50 mM CaCl_2_ solution [CaCl_2_ solution:0.6 M mannitol, 50 mM CaCl_2_, 1 g/l BSA, 5 mM HEPES, and 40 mM glycine, pH 5.7]. After 30 minutes in the CaCl_2_ solution, we rinsed the beads one time in 15 ml of FW solution. We then transferred the alginate-embedded protoplasts into 60-ml Nalgene jars containing 2.5 ml of liquid 0.4 M Pic/TDZ medium supplemented with 1.0 mM putrescene, 0.1 mM spermidine, 1.0 mM spermine, 40 mM glycine, 5 mM L-arginine, 5 mM L-leucine, 5 mM L-lysine, 0.6 mM ascorbic acid, 0.7 mM citric acid, 0.1 mM reduced glutathione, 0.8 mM L-cysteine, and 0.5 ml of a 7-day-old 1103P suspension culture conditioned to grow in 0.4 M Pic/TDZ (feeder suspension). We incubated the suspension in the dark at 50 rpm and 25°C. After 14 days, we added 3 ml of Pic/TDZ medium with the supplements listed above, but without mannitol [0.0 M Pic/TDZ medium], thereby reducing the starting mannitol concentration to 0.2 M. After an additional 14 days, we removed 3 ml of the suspension cultures from the jars and replaced it with 3 ml of 0.0 M Pic/TDZ medium, thereby reducing the starting mannitol concentration to 0.1 M.

### Plant regeneration from protoplast-derived callus

Once protoplasts developed into callus colonies of ~16–32 cells within the alginate beads (~42–70 days), we manually transferred individual beads to a 100 × 20-mm petri dish containing 20 ml WPM minimal organics medium [WPM medium supplemented with 20 g/l sucrose, 1 g/l casein, 1 mM MES, without hormones or activated charcoal]. We repeated this transfer/washing process three times to eliminate any of the feeder suspension cells. We transferred 25 beads containing large calli and embryos to 100 ×20-mm petri dishes containing 40 ml of agar solidified WPM medium supplemented with 20 g/l sucrose, 1 g/l casein, 1 mM MES, 500 mg/l activated charcoal, 2.2 μM BAP, 0.55 μM 1-Naphthaleneacetic acid (NAA) and incubated under continuous light, where embryos germinated out of the gel matrix**.** After germination, we transferred individual seedlings to Phytatray™ II (Sigma product No. P5929) containing agar-solidified WPM medium supplemented with 20 g/l sucrose, 1 g/l casein, 1 mM MES, 500 mg/l activated charcoal, and 0.05 μM indole-butyric acid (IBA), where they developed into whole plants. We acclimated plants to soil in a growth chamber for 4 weeks, and then transferred them to a greenhouse.

### CRISPR-Cas9 plasmid cloning

For editing experiments using plasmid DNA containing the CRISPR-Cas9 components, we used the vector pDIRECT_10E designed by the Daniel Voytas group [33], which was obtained from Addgene (Plasmid #91209). This vector contains a 35S_pro_:Cas9 cassette and allows one to clone one or multiple gRNAs under a single CmYLCV promoter. Multiple gRNAs are post-transcriptionally processed through the tRNA approach [34]. To target *VvPDS* genes, we cloned two gRNAs previously published targeting regions in exon fifth and seventh [11, 19]. To target DELLA domain in *VvGAI* gene, we designed 6 gRNAs, which were cloned in three vectors, as gRNA1, 3, and 5 in vector #1; gRNA2, 4, and 6 in vector #2; and gRNA1 and 6 in vector #3. The cloning of the gRNAs was done by Golden Gate method following the protocol developed by the Voytas group [33]. The sequences of the primers used to generate the gRNAs are indicated in Table S4.

### CRISPR-Cas9 editing by plasmid transfection in grapevine protoplast

For protoplast transfections, we isolated and purified protoplasts as described above. We washed the protoplasts by centrifugation at 500 g for 8 minutes and resuspending them in 4 ml of W5 solution [154 mM NaCl, 125 mM CaCl_2_, 5 mM KCl, 2 mM MES, pH 5.7] in a 15-ml tube. We repeated this centrifugation process, resuspended the protoplasts in 3 ml of W5 solution and held on ice. After 30 minutes, we pelleted the protoplasts by centrifugation at 500 g for 8 minutes, resuspended in MMG solution [4 mM MES, 0.4 M Mannitol, 15 mM MgCl_2,_ pH 5.7], at a density of 5 × 10^6^ cells per ml. We transferred 200 μl (1 × 10^6^ cells) of that solution to 1.5-ml tubes. We pelleted the protoplasts at 500 × g for 8 minutes and removed 150 μl of MMG supernatant. We added 10–20 μg of the CRISPR plasmid DNA directly to the protoplasts using a gentle swirling motion, followed by 200 μl (plus the volume of the plasmid DNA added) of a freshly prepared PEG solution [40% w/v PEG 4000 Sigma No. 95904, 0.2 M mannitol, 0.1 M CaCl_2_]. Next, we added 150 μl of MMG solution and gently mixed the contents of the tube. We incubated the transfection mix for 15 minutes at room temperature in the dark, and then stopped the reaction by adding 880 μl of W5 solution to each tube and inverting the tube gently to mix.

We transferred the transfected protoplasts to a 15-ml centrifuge tube and added 3 ml of W5. We pelleted the protoplasts at 500 g for 8 minutes and resuspended in 4 ml of FW solution. This washing step was repeated 3 times to remove the presence of calcium, which would prevent the sodium alginate from remaining fluid. Finally, the protoplasts were resuspended in 1 ml of FW solution and 1 ml of SAS, and alginate beads were prepared and cultured in 60-ml Nalgene jars as described above.

Once the dividing cells reached the 16- to 32-cells stage, we removed the beads from liquid cultures, washed 3 times in WPM minimal organics medium and plated onto agar-solidified plates to induce embryo formation as indicated above. ***Note:*** For each set of experiments, we ran controls consisting of non-treated protoplasts, which were prepared as the transfected protoplasts, but not treated with plasmid DNA.

### CRISPR-Cas9 editing by RNPs transfection in grapevine protoplast

We generated RNP complexes using Cas9 protein (CAS9PROT-50UG, Sigma) and synthetic gRNAs (Sigma). We ensembled RNP complexes *in vitro* by mixing 2.5 μl of 10 μM Cas9 protein solution (25 pmol of Cas9) with 2.5 μl of 30 μM gRNA solution (75 pmol of gRNA) and incubating the mix for 10–15 minutes at room temperature. We prepared protoplasts as described for plasmid transfection, but we adjusted their density to 1 × 10^7^ protoplast per ml in MMG. We added 100 μl (1 × 10^6^ cells) of that solution to 5 μl RNP mix followed immediately by an equal volume of PEG 4000. We gently inverted the tubes to mix and incubated at room temperature in the dark. After 15–20 minutes, we stopped the transfections by adding 880 μl of W5 solution to each tube and inverting the tube gently to mix, and then washed the protoplasts as described above prior to encapsulating in alginate beads. Alginate bead cultures, embryo formation, and whole-plant regeneration was done as described above.

### Checking CRISPR activity

To check CRISPR activity, we collected root or leaf samples from plants regenerated from transfected protoplasts. We purified genomic DNA using the commercial DNeasy kit (Qiagen), according to the manufacturer’s protocol. The detection of genome editing events was done by amplicon sequencing. A detailed protocol is provided at Zhang, J. 2022 [35]. Briefly, we performed polymerase chain reactions (PCRs) with primers flanking the regions targeted by the different gRNAs. The sequences of the primers used are indicated in Table S4. Then, we added barcoded adaptors through a second nested PCR, pooled the PCR products, purified, and subjected them to CRISPR sequencing using the sequencing services provided by MGH CCIB DNA Core (https://dnacore.mgh.harvard.edu/new-cgi-bin/site/pages/crispr_sequencing_main.jsp).

## Acknowledgements

This research was financially supported by the CDFA Pierce’s Disease and Glassy-winged Sharpshooter Board. We thank Mariana Padilla for excellent technical support.

## Author Contributions

D.M.T. contributed to development of the protoplast isolation, purification, transfection, and plant regeneration protocols, funding acquisition, project administration, experiment designing and execution, and manuscript writing and editing. J.M.D. contributed to gene identification, vector design and construction, data analysis, and manuscript writing and editing.

## Data Availability

The authors confirm that all the experimental data are available and accessible via the main text and/or the supplementary information.

## Conflict of Interests

D.M.T. is inventor in a US patent application filed by the University of California on the protoplast regeneration system described in this study.

## Supplementary Data

Supplementary data is available at *Horticulture Research* online.

## Supplementary Material

Web_Material_uhad266
