# Supplementary material for: An efficient protoplast-based genome editing protocol for Vitis species
Source: Hortic Res. 2023 Dec 13;11(1):uhad266. doi: 10.1093/hr/uhad266 (PMC11184525; doi:10.1093/hr/uhad266)
Supplement: Web_Material_uhad266 [file web_material_uhad266.zip › FigureS2 (002).pdf]

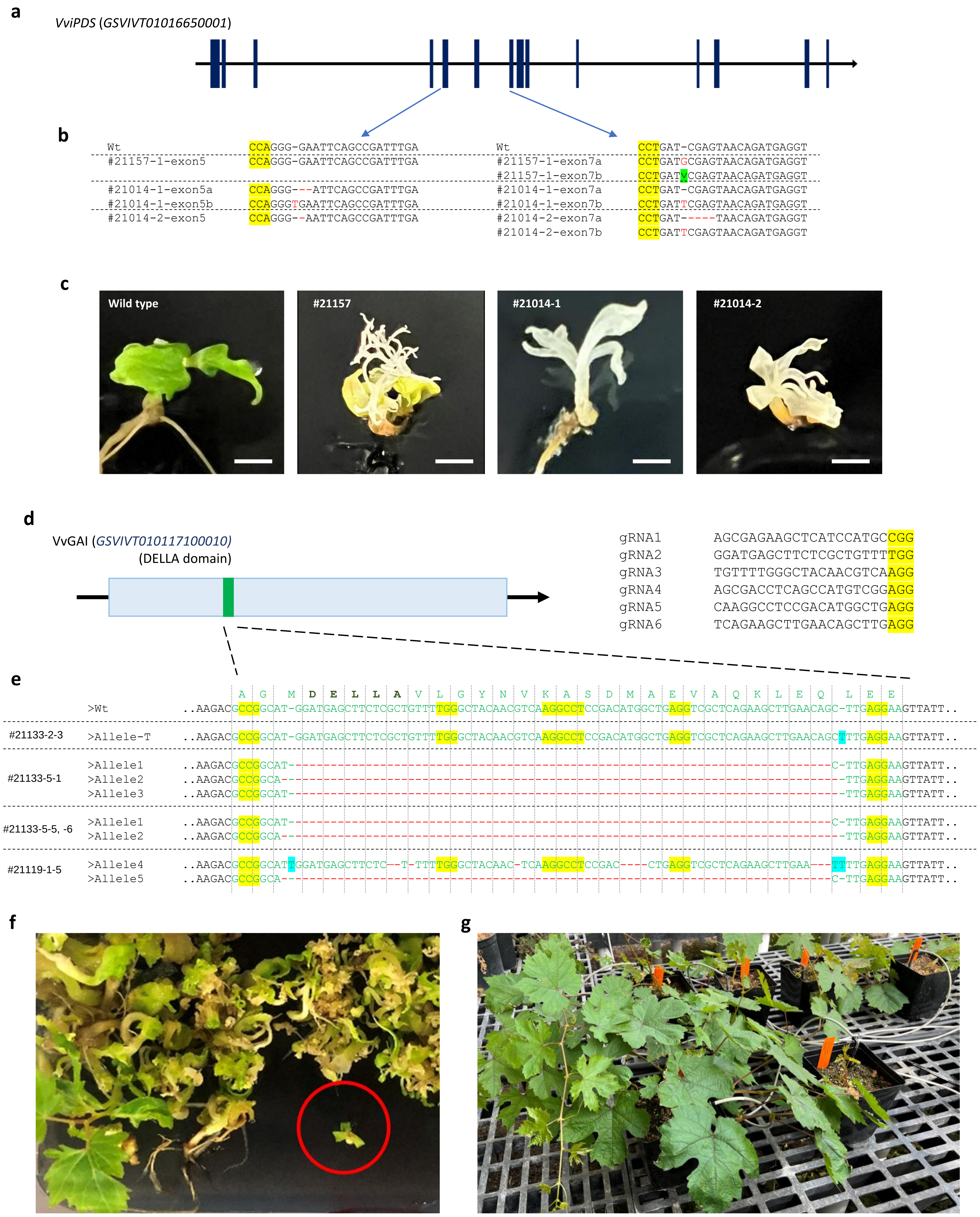
a
VviPDS (GSVIVT01016650001)
b
Wt
#21157-1-exon5
#21014-1-exon5a
#21014-1-exon5b
#21014-2-exon5
CCAGGG-GAATTCAGCCGATTTGA
CCAGGG-GAATTCAGCCGATTTGA
CCAGGG---ATTCAGCCGATTTGA
CCAGGGTGAATTCAGCCGATTTGA
CCAGGG--AATTCAGCCGATTTGA
Wt
#21157-1-exon7a
#21157-1-exon7b
#21014-1-exon7a
#21014-1-exon7b
#21014-2-exon7a
#21014-2-exon7b
CCTGAT-CGAGTAACAGATGAGGT
CCTGATGCGAGTAACAGATGAGGT
CCTGATYCGAGTAACAGATGAGGT
CCTGAT-CGAGTAACAGATGAGGT
CCTGATTCGAGTAACAGATGAGGT
CCTGAT-----TAACAGATGAGGT
CCTGATTCGAGTAACAGATGAGGT
c
Wild type
#21157
#21014-1
#21014-2
d
VvGAI (GSVIVT010117100010)
(DELLA domain)
gRNA1 AGCGAGAAGCTCATCCATGCCGG
gRNA2 GGATGAGCTTCTCGCTGTTTTGG
gRNA3 TGTTTTGGGCTACAACGTCAAGG
gRNA4 AGCGACCTCAGCCATGTCGGAGG
gRNA5 CAAGGCCTCCGACATGGCTGAGG
gRNA6 TCAGAAGCTTGAACAGCTTGAGG
e
A G M D E L L A V L G Y N V K A S D M A E V A Q K L E Q L E E
>Wt
#21133-2-3 >Allele-T
#21133-5-1 >Allele1 >Allele2 >Allele3
#21133-5-5, -6 >Allele1 >Allele2
#21119-1-5 >Allele4 >Allele5
f
g
