# Supplementary material for: An efficient protoplast-based genome editing protocol for Vitis species
Source: Hortic Res. 2023 Dec 13;11(1):uhad266. doi: 10.1093/hr/uhad266 (PMC11184525; doi:10.1093/hr/uhad266)
Supplement: Web_Material_uhad266 [file web_material_uhad266.zip › FigureS3.pdf]

Wt	AGCCAGGG-GAATTCAGCCGATTTGA
**Colombard**	
#31	AGCCAGGGTGAATTCAGCCGATTTGA
	AGCCAGGG--AATTCAGCCGATTTGA
#2, 4	AGCCAGGGGGAATTCAGCCGATTTGA
	AGCCAGGG---ATTCAGCCGATTTGA
#41	AGCCAGGGTGAATTCAGCCGATTTGA
	AGCCAGGG------CAGCCGATTTGA
#17	AGCCAGGGGGAATTCAGCCGATTTGA
	AGCCAGGG------CAGCCGATTTGA
#18	AGCCAGGGTGAATTCAGCCGATTTGA
	AGCCAGGG--------GCCGATTTGA
#19	AGCCAGGG---ATTCAGCCGATTTGA
	AGCCAGGG-----TCAGCCGATTTGA
#39	AGCCAGGG--AATTCAGCCGATTTGA
	AGCCAGG------TCAGCCGATTTGA
#43	AGCCAGGGAGAATTCAGCCGATTTGA
	del 70bp
#30,42	AGCCAGGGTGAATTCAGCCGATTTGA
#29	AGCCAGGG---ATTCAGCCGATTTGA
#6,7,45	AGCCAGGG-------------TTTGA
V. arizonica	
#4-1,4-2	---------------AGCCGATTTGA
	del28--G-GAATTCAGCCGATTTGA
