# Supplementary figures and images for: An efficient protoplast-based genome editing protocol for Vitis species
Source: Hortic Res. 2023 Dec 13;11(1):uhad266. doi: 10.1093/hr/uhad266 (PMC11184525; doi:10.1093/hr/uhad266)

Rootstock 101-14

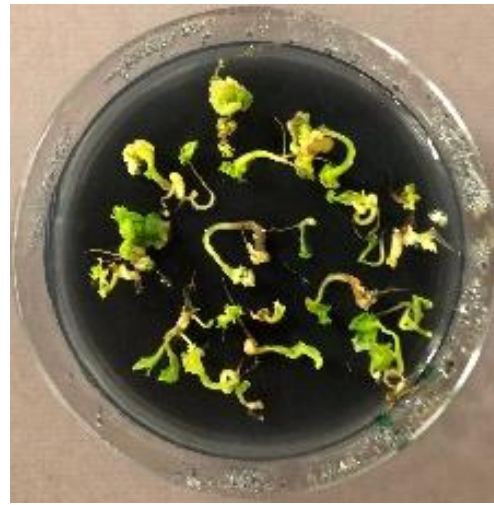

Rootstock GRN1

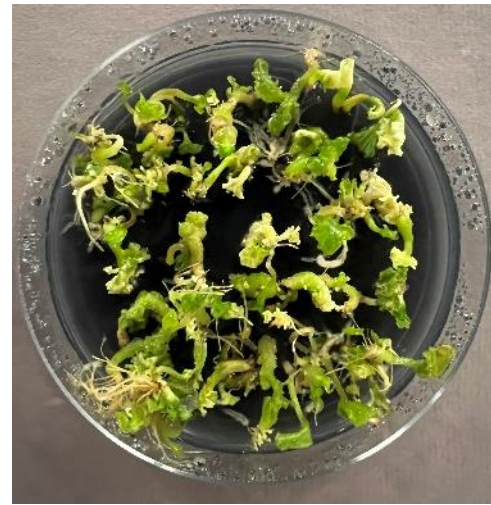

*Vitis arizonica*

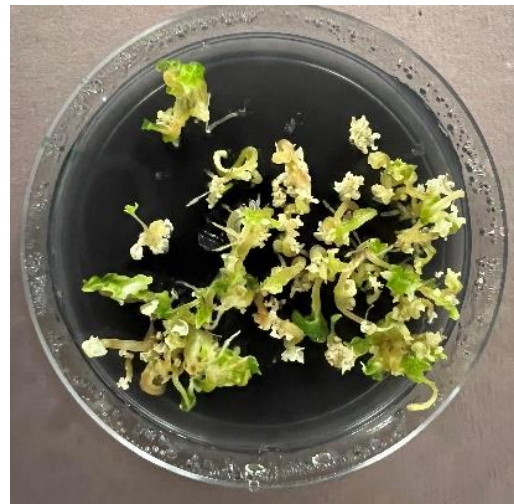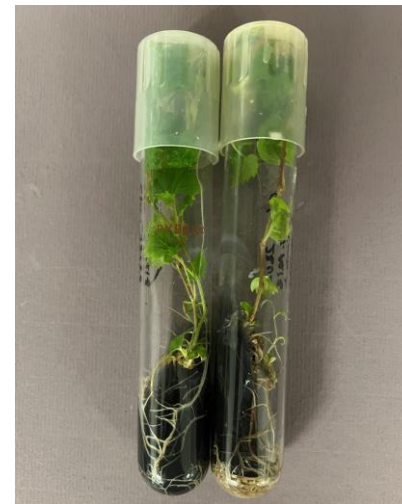

Supplement: Web_Material_uhad266 [file web_material_uhad266.zip › FigureS1.pdf]
